# Implementation of multilayer perceptron network with highly uniform passive memristive crossbar circuits

**DOI:** 10.1038/s41467-018-04482-4

**Published:** 2018-06-13

**Authors:** F. Merrikh Bayat, M. Prezioso, B. Chakrabarti, H. Nili, I. Kataeva, D. Strukov

**Affiliations:** 1 Electrical and Computer Engineering Department, University of California, Santa Barbara, CA 93117 USA; 2 DENSO CORP, 500-1 Minamiyama, Komenoki-cho, Nisshin 470-0111 Japan

## Abstract

The progress in the field of neural computation hinges on the use of hardware more efficient than the conventional microprocessors. Recent works have shown that mixed-signal integrated memristive circuits, especially their passive (0T1R) variety, may increase the neuromorphic network performance dramatically, leaving far behind their digital counterparts. The major obstacle, however, is immature memristor technology so that only limited functionality has been reported. Here we demonstrate operation of one-hidden layer perceptron classifier entirely in the mixed-signal integrated hardware, comprised of two passive 20 × 20 metal-oxide memristive crossbar arrays, board-integrated with discrete conventional components. The demonstrated network, whose hardware complexity is almost 10× higher as compared to previously reported functional classifier circuits based on passive memristive crossbars, achieves classification fidelity within 3% of that obtained in simulations, when using ex-situ training. The successful demonstration was facilitated by improvements in fabrication technology of memristors, specifically by lowering variations in their *I–V* characteristics.

## Abstract (web summary)

Memristive devices used in neuromorphic computing typically need to be accessed using transistors, adding circuit complexity and size. In this work, the authors demonstrate a neural network using a transistor-free passive memristor crossbar array, offering potential circuit miniaturisation and energy savings.

## Introduction

Started more than half a century ago, the field of neural computation has known its ups and downs, but since 2012, it exhibits an unprecedented boom triggered by the dramatic breakthrough in the development of deep convolutional neuromorphic networks^1,2^. The breakthrough^3^ was enabled not by any significant algorithm advance, but rather by the use of high performance graphics processors^4^, and the further progress is being fueled now by the development of even more powerful graphics processors and custom integrated circuits^5–7^. Nevertheless, the energy efficiency of these implementations of convolutional networks (and other neuromorphic systems^8–11^) remains well below that of their biological prototypes^12,13^, even when the most advanced CMOS technology is used. The main reason for this efficiency gap is that the use of digital operations for mimicking biological neural networks, with their high redundancy and intrinsic noise, is inherently unnatural. On the other hand, recent works have shown^11–16^ that analog and mixed-signal integrated circuits, especially using nanoscale devices, may increase the neuromorphic network performance dramatically, leaving far behind both their digital counterparts and biological prototypes and approaching the energy efficiency of the brain. The background for these advantages is that in such circuits the key operation performed by any neuromorphic network, the vector-by-matrix multiplication, is implemented on the physical level by utilization of the fundamental Ohm and Kirchhoff laws. The key component of this circuit is a nanodevice with adjustable conductance *G*—essentially an analog nonvolatile memory cell—used at each crosspoint of a crossbar array, and mimicking the biological synapse.

Though potential advantages of specialized hardware for neuromorphic computing had been recognized several decades ago^17,18^, up until recently, adjustable conductance devices were mostly implemented using the standard CMOS technology^13^. This approach was used to implement several sophisticated, efficient systems—see, e.g., refs.^14,15^. However, these devices have relatively large areas leading to higher interconnect capacitance and hence larger time delays. Fortunately, in the last decade, another revolution has taken place in the field of nanoelectronic memory devices. Various types of emerging nonvolatile memories are now being actively investigated for their use in fast and energy-efficient neuromorphic networks^19–41^. Of particular importance, is the development of the technology for programmable, nonvolatile two-terminal devices called ReRAM or memristors^42,43^. The low-voltage conductance *G* of these devices may be continuously adjusted by the application of short voltage pulses of higher, typically >1 V amplitude^42^. These devices were used to demonstrate first neuromorphic network providing pattern classification^21,26,28,30,32,40^. The memristors can have a very low chip footprint, which is determined only by the overlap area of the metallic electrodes, and may be scaled down below 10 nm without sacrificing their endurance, retention, and tuning accuracy, with some of the properties (such as the ON/OFF conductance ratio) being actually improved^44^.

Much of the previous very impressive demonstrations of neuromorphic networks based on resistive switching memory devices, including pioneering work by IBM^25,34^, were based on the so-called 1T1R technology, in which every memory cell is coupled to a select transistor^22,27–31^. The reports of neuromorphic functionality based on passive 0T1R or 1D1R circuits (in which acronyms stand for 0 Transistor or 1 Diode +1 Resistive switching device per memory cell, respectively) have been so far very limited^26,39^, in part due to much stricter requirement for memristors’ *I–V* uniformity for successful operation. The main result of this paper is the experimental demonstration of a fully functional, board-integrated, mixed-signal neuromorphic network based on passively integrated metal-oxide memristive devices. Our focus on 0T1R memristive crossbar circuits is specifically due to their better performance and energy-efficiency prospects, which can be further improved by three-dimensional monolithical integration^45–47^. Due to the extremely high effective integration density, three-dimensional memristive circuits will be instrumental in keeping all the synaptic weights of a large-scale artificial neural networks locally, thus cutting dramatically the energy and latency overheads of the off-chip communications. The demonstrated network is comprised of almost an order of magnitude higher number of devices as compared to the previously reported neuromorphic classifiers based on passive crossbar circuits^26^. The inference, the most common operation in applications of deep learning, is performed directly in a hardware, which is different from many previous works that relied on post-processing the experimental data with external computer to emulate the functionality of the whole system^25–27,34,39,40^.

## Results

### Integrated memristors

**Fig. 1:**
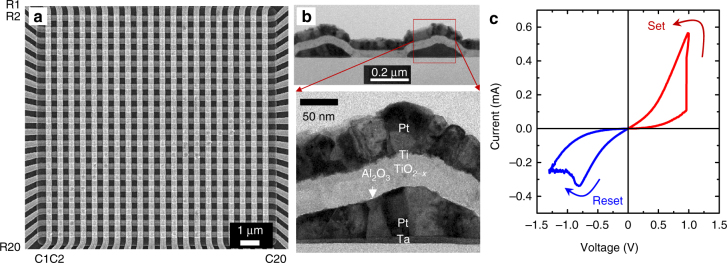
Passive memristive crossbar circuit. **a** A top-view SEM and **b** cross-section TEM images of 20 × 20 Pt/Al_2_O_3_/TiO_2−*x*_/Ti/Pt crossbar circuit; **c** A typical *I–V* switching curve

The passive 20 × 20 crossbar arrays with Pt/Al_2_O_3_/TiO_2−*x*_/Ti/Pt memristor at each crosspoint were fabricated using a technique similar to that reported in ref. ^26^ (Fig. 1). Specifically, the bilayer binary oxide stack was deposited using low temperature reactive sputtering method. The crossbar electrodes were evaporated using oblique angle physical vapor deposition (PVD) and patterned by lift-off technique using lithographical masks with 200-nm lines separated by 400-nm gaps. Each crossbar electrode is contacted to a thicker (Ni/Cr/Au 400 nm) metal line/bonding pad, which are formed at the last step of the fabrication process. As evident from Fig. 1a, b, due to the utilized undercut in the photoresist layer and tilted PVD sputtering in the lift-off process, the metal electrodes have roughly triangular shape with ~250 nm width. Such shape of the bottom electrodes ensured better step coverage for the following processing layers and, in particular, helped to reduce the top electrode resistance. The externally measured (pad-to-pad) crossbar line resistance for the bonded chip is around 800 Ω. It is similar to that of smaller crossbar circuit reported in ref.^26^ due to the dominant contribution of the contact between crossbar electrode and thicker bonding lines.

**Fig. 2:**
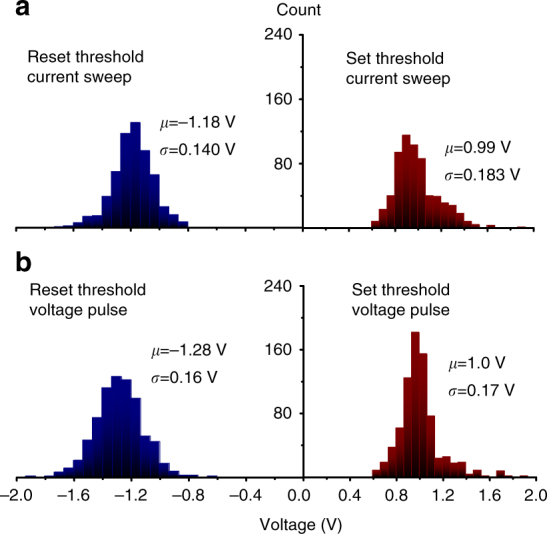
Set and reset threshold statistics. The data are shown for seven 20 × 20- device crossbar arrays at memristor switching with **a** current and **b** voltage pulses. The set/reset thresholds are defined as the smallest voltages at which the device resistance is increased/decreased by >5% at the application of a voltage or current pulse of the corresponding polarity. The legends show the corresponding averages and standard deviations for the switching threshold distributions. Note that the variations are naturally better when only considering devices within a single crossbar circuit, and in addition, excluding memristors at the edges of the circuit, which typically contribute to the long tails of the histograms. For example, excluding these devices, *µ* is 1.0 V/−1.2 V and *σ* is 0.13 V/0.15 V for voltage controlled set/reset for one of the crossbars used in the experiment

Majority of the devices required an electroforming step which consisted of one-time application of a high current (or voltage) ramp bias. We have used both increasing amplitude current and voltage sweeps for forming but did not see much difference in the results of the forming procedure (Fig. 2). This could be explained by the dominant role of capacitive discharge from the crossbar line during forming, which cannot be controlled well by external current source or current compliance. The devices were formed one at a time, and to speed up the whole process, an automated setup has been developed—see Methods section for more details. The setup was used for early screening of defective samples and has allowed a successful forming and testing of numerous crossbar arrays (Fig. 2). Specially, about 1–2.5% of the devices in the crossbar arrays, i.e., 10 or less out of 400 total, could not be formed with the algorithm parameters that we used. (It might have been possible to form even these devices by applying larger stress but we have not tried it in this experiment to avoid permanently damaging the crossbar circuit.) Typically, the failed devices were stuck at some conductance state, comparable to the range of conductances utilized in the experiment, and as a result have negligible impact on the circuit functionality.

Memristor *I–V* characteristics are nonlinear (Fig. 1c) due to the alumina barrier between the bottom electrode and the switching layer. *I–V*’s nonlinearity provides sufficient selector functionality to limit leakage currents in the crossbar circuit, and hence reduce disturbance of half-selected devices during conductance tuning. It is worth mentioning that the demonstrated nonlinearity is weaker as compared to state-of-the-art selector devices that are developed in the context of memory applications. However, our analysis (Supplementary Note 1) shows that strengthening *I–V* nonlinearity would only reduce power consumption during very infrequent tuning operation but otherwise have no impact on the more common inference operation in the considered neuromorphic applications.

**Fig. 3:**
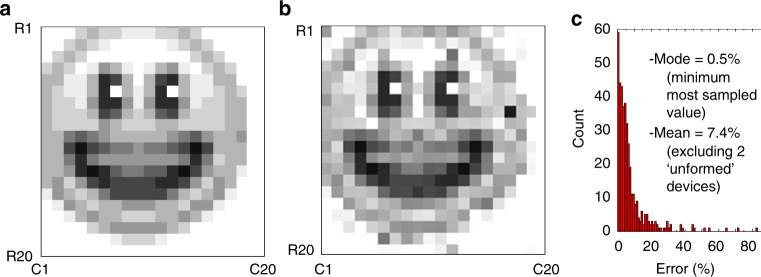
High precision tuning. **a** The desired “smiley face” pattern, quantized to 10 gray levels. **b** The actual resistance values measured after tuning all devices in 20 × 20 memristive crossbar with the nominal 5% accuracy, using the automated tuning algorithm^48^, and **c** the corresponding statistics of the tuning errors, which is defined as normalized absolute difference between the target and actual conductance values. On panel **a**, the white/black pixels correspond to 96.6 KΩ/7 KΩ, measured at 0.2 V bias. The tuning was performed with 500-µs-long voltage pulses with amplitudes in a [0.8 V, 1.5 V]/[−1.8 V, −0.8 V] range to increase/decrease device conductance. (Supplementary Fig. 3 shows absolute values of resistances and absolute error for the data on panels **b** and **c**, respectively)

Most importantly, memristive devices in the fabricated 20 × 20 crossbar circuits have uniform characteristics with gradual (analog) switching. The distributions of the effective set and reset voltages are sufficiently narrow (Fig. 2) to allow precise tuning of devices’ conductances to the desired values in the whole array (Fig. 3, Supplementary Fig. 12), which is especially challenging in the passive integrated circuits due to half-select disturbance. For example, an analog tuning was essential for other demonstrations based on passive memristive circuits, though was performed with much cruder precision^19,39^. A comparable tuning accuracy was demonstrated in ref. ^40^, though for less dense but much more robust to variations 1T1R structures, in which each memory cell is coupled with a dedicated low-variation transistor. Furthermore, memristors can be retuned multiple times without noticeable aging—see Supplementary Note 2 for more details.

### Multilayer perceptron implementation

**Fig. 4:**
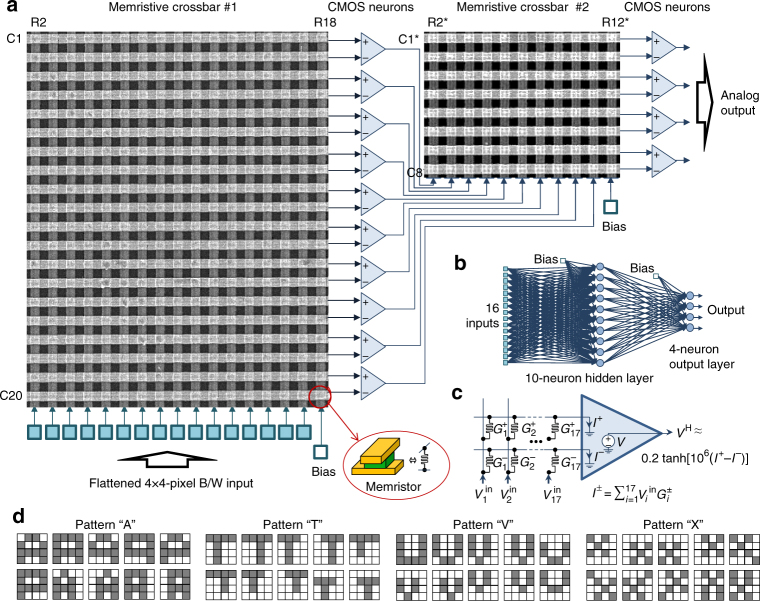
Multilayer perceptron classifier. **a** A perceptron diagram showing portions of the crossbar circuits involved in the experiment. **b** Graph representation of the implemented network; **c** Equivalent circuit for the first layer of the perceptron. For clarity, only one hidden layer neuron is shown; **d** A complete set of training patterns for the 4-class experiment, stylistically representing letters “A”, “T”, “V” and “X”

Two 20 × 20 crossbar circuits were packaged and integrated with discrete CMOS components on two printed circuit boards (Supplementary Fig. 2b) to implement the multilayer perceptron (MLP) (Fig. 4). The MLP network features 16 inputs, 10 hidden-layer neurons, and 4-outputs, which is sufficient to perform classification of 4 × 4-pixel black-and-white patterns (Fig. 4d) into 4 classes. With account of bias inputs, the implemented neural network has 170 and 44 synaptic weights in the first and second layers, respectively.

The integrated memristors implement synaptic weights, while discrete CMOS circuitry implements switching matrix and neurons. Each synaptic weight is implemented with a pair of memristors, so that 17 × 20 and 11 × 8 contiguous subarrays were involved in the experiment (Fig. 4a), i.e., almost all of the available memristors in the first crossbar and about a quarter of the devices in the second one. The switching matrix was implemented with analog discrete component multiplexers and designed to operate in two different modes. The first one is utilized for on-board forming of memristors as well as their conductance tuning during weight import. In this operation mode, the switching matrix allows the access to any selected row and column and, simultaneously, the application of a common voltage to all remaining (half-selected) crossbar lines, including an option of floating them. The voltages are generated by an external parameter analyzer. In the second, inference mode the switching matrix connects the crossbar circuits to the neurons as shown in Fig. 4a and enables the application of ±0.2 V inputs, corresponding to white and black pixels of the input patterns. Concurrently, the measurement of output voltages of the perceptron network is carried out. The whole setup is controlled by a general-purpose computer (Supplementary Fig. 2c).

The neuron circuitry is comprised of three distinct stages (Supplementary Fig. 2a). The first stage consists of inverting operational amplifier, which maintains a virtual ground on the crossbar row electrodes. Its voltage output is a weighted sum between the input voltages, applied to crossbar columns (Fig. 4a), and the conductances of the corresponding crosspoint devices. The second stage op-amp computes the difference between two weighted sums calculated for the adjacent line of the crossbar. The operational amplifier’s output in this stage is allowed to saturate for large input currents, thus effectively implementing tanh-like activation function. In the third and final stage of the neuron circuit, the output voltage is scaled down to be within −0.2 V to +0.2 V range before applying it to the next layer. The voltage scaling is only implemented for the hidden layer neurons to ensure negligible disturbance of the state of memristors in the second crossbar array.

With such implementation, perceptron operation for the first and second layers is described by the following equations:

1 $$V_j^{\mathrm{H}} \approx 0.2\;{\mathrm{tanh}}\left[ {10^6\left( {I_j^ + - I_j^ - } \right)} \right],\hskip2pt I_j^ \pm = \mathop {\sum }\limits_{i = 1}^{17} V_i^{{\mathrm{in}}}G_{ij}^{(1) \pm }$$

2 $$V_k^{{\mathrm{out}}} \approx 10^6\left( {I_k^ + - I_k^ - } \right),\hskip2pt I_k^ \pm = \mathop {\sum }\limits_{j = 1}^{11} V_j^{\mathrm{H}}G_{jk}^{(2) \pm }$$

Here *V* ^in^, *V* ^H^, *V* ^out^ are, respectively, perceptron input, hidden layer output, and perceptron output voltages. *G*^(1)±^ and *G*^(2)±^ are the device conductances in the first and second crossbar circuits, with ± superscripts denoting a specific device of a differential pair, while *I*^±^ are the currents flowing into the corresponding neurons. *j* and *k* are hidden and output neuron indexes, while *i* is the pixel index of an input pattern. The additional bias inputs *V*_17_^in^ and *V*_11_^H^ are always set to +0.2 V.

### Pattern classification

In our first set of experiments, the multilayer perceptron was trained ex-situ by first finding the synaptic weights in the software-implemented network, and then importing the weights into the hardware. Because of limited size of the classifier, we have used custom 4-class benchmark, which is comprised of a total of 40 training (Fig. 4d) and 640 test (Supplementary Fig. 4) 4 × 4-pixel black and white patterns representing stylized letters “A”, “T”, “V”, and “X”. As Supplementary Fig. 5 shows, the classes of the patterns in the benchmark are not linearly separable and the use of multi-bit (analog) weights significantly improve performance for the implemented training algorithm.

In particular, the software-based perceptron was trained using conventional batch-mode backpropagation algorithm with mean-square error cost function. The neuron activation function was approximated with tangent hyperbolic with a slope specific to the hardware implementation. We assumed a linear *I–V* characteristics for the memristors, which is a good approximation for the considered range of voltages used for inference operation (Fig. 1c). During the training the weights were clipped within (10 μS, 100 μS) conductance range, which is an optimal range for the considered memristors.

In addition, two different approaches for modeling weights were considered in the software network. In the simplest, hardware-oblivious approach, all memristors were assumed to be perfectly functional, while in a more advanced, hardware-aware approach, the software model utilized additional information about the defective memristors. These were the devices whose conductances were experimentally found to be stuck at some values, and hence could not be changed during tuning.

**Fig. 5:**
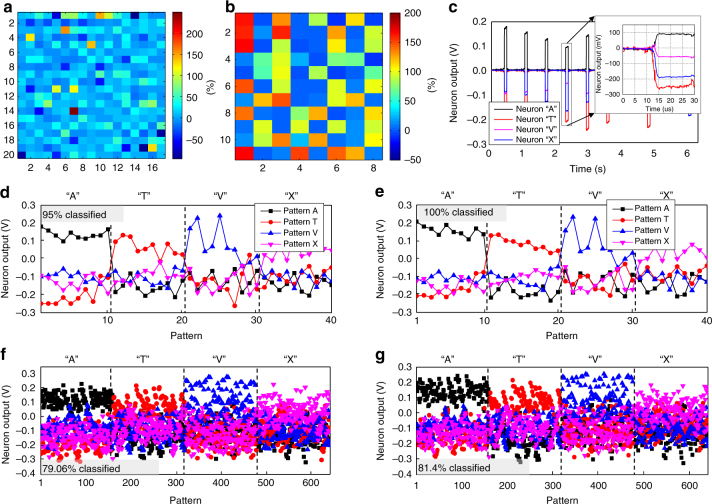
Ex-situ training experimental results. **a**, **b** The normalized difference between the target and the actual conductances after tuning in **a** the first and **b** the second layer of the network for the hardware-oblivious training approach; **c** Time response of the trained network for 6 different input patterns, in particular showing less than 5 μs propagation delay. Perceptron output voltage for **d**, **f** hardware-oblivious and **e**, **g** hardware-aware ex-situ training approaches, with **d**-**g** panels showing measured results for training/test patterns

The calculated synaptic weights were imported into the hardware by tuning memristors’ conductances to the desired values using an automated write-and-verify algorithm^48^. The stuck devices were excluded from tuning for the hardware-aware training approach. To speed up weight import, the maximum tuning error was set to 30% of the target conductance (Fig. 5a, b), which is adequate import precision for the considered benchmark according to the simulation results (Supplementary Fig. 5). Even though tuning accuracy was often worse than 30%, the weight errors were much smaller and, e.g., within 30% for 42 weights (out of 44 total) in the second layer of the network (Supplementary Fig. 6). This is due to our differential synapses implementation, in which one of the conductances was always selected to have the smallest (i.e., 10 µS) value and the cruder accuracy was used for tuning these devices because of their insignificant contribution to the actual weight.

After weight import had been completed, the inference was performed by applying ±0.2 V inputs specific to the pattern pixels and measuring four analog voltage outputs. Figure 5c shows typical transient response. Though the developed system was not optimized for speed, the experimentally measured classification rate was quite high—about 300,000 patterns per second and was mainly limited by the chip-to-chip propagation delay of analog signals on the printed circuit board.

Figure 5d, e shows classification results for the considered benchmark using the two different approaches. (In both software simulations and hardware experiments, the winning class was determined by the neuron with maximum output voltage.) The generalization functionality was tested on a 640 noisy test patterns (Supplementary Fig. 4), obtained by flipping one of the pixels in the training images (Fig. 4d). The experimentally measured fidelity on a training and test set patterns for the hardware-oblivious approach were 95% and 79.06%, respectively (Fig. 5d, f), as compared to 100% and 82.34% achieved in the software (Supplementary Fig. 5). As expected, the experimental results were much better for hardware-aware approach, i.e., 100% for the training patterns and 81.4% for the test ones (Fig. 5e, g).

It should be noted that the achieved classification fidelity on test patterns is far from ideal 100% value due to rather challenging benchmark. In our demonstration, the input images are small and addition of noise, by flipping one pixel, resulted in many test patterns being very similar to each other. In fact, many of them are very difficult to classify even for a human, especially distinguishing between test patterns ‘V’ and ‘X’.

In our second set of experiments, we have trained the network in-situ, i.e., directly in a hardware^21^. (Similar to our previous work^26^, only inference stage was performed in a hardware during such in-situ training, while other operations, such as computing and storing the necessary weight updates, were assisted by an external computer.) Because of limitations of our current experimental setup, we implemented in-situ training using fixed-amplitude training pulses, which is similar to Manhattan rule algorithm. The classification performance for this method was always worse as compared to that of both hardware-aware and hardware-oblivious ex-situ approaches. For example, the experimentally measured fidelity for 3-pattern classification task was 70%, as compared to 100% classification performance achieved on training set using both ex-situ approaches. This is expected because in ex-situ training the feedback from read measurements of the tuning algorithm allows to effectively cope with switching threshold variations by uniquely adjusting write pulse amplitude for each memristor, which is not the case for the fixed-amplitude weight update (Supplementary Fig. 7). We expect that fidelity of in-situ trained network can be further improved using variable-amplitude implementation^49^.

## Discussion

We believe that the presented work is an important milestone towards implementation of extremely energy efficient and fast mixed-signal neuromorphic hardware. Though demonstrated network has rather low complexity to be useful for practical applications, it has all major features of more practical large-scale deep learning hardware—a nonlinear neuromorphic circuit based on metal-oxide memristive synapses integrated with silicon neurons. The successful board-level demonstration was mainly possible due to the advances in memristive circuit fabrication technology, in particular much improved uniformity and reliability of memristors.

Practical neuromorphic hardware should be able to operate correctly under wide temperature ranges. In the proposed circuits, the change in memristor conductance with ambient temperature (Supplementary Fig. 9) is already partially compensated by differential synapse implementation. Furthermore, the temperature dependence of *I–V* characteristics is weaker for higher conductive states (Supplementary Fig. 9). This can be exploited to improve robustness with respect to variations in ambient temperature, for example, by setting the device conductances within a pair to *G*_BIAS_ ± *G*/2, where *G*_BIAS_ is some large value. An additional approach is to utilize memristor, with conductance *G*_M_, in the feedback of the second operational amplifier stage of the original neuron circuit (Supplementary Fig. 2a). In this case, the output of the second stage is proportional to Σ_*i*_*V*_*i*_^in^(*G*_*i*_^+^-*G*_*i*_^−^)/*G*_M_ with temperate drift further compensated assuming similar temperature dependence for the feedback memristor.

Perhaps the only practically useful way to scale up the neuromorphic network complexity further is via monolithical integration of memristors with CMOS circuits. Such work has already been started by several groups^19,30^, including ours^47^. We envision that the most promising implementations will be based on passive memristor technology, i.e., similar to the one demonstrated in this paper, because it is suitable for monolithical back-end-of-line integration of multiple crossbar layers^46^. The three dimensional nature of such circuits^50^ will enable neuromorphic networks with extremely high synaptic density, e.g., potentially reaching 10^13^ synapses in one square centimeter for 100-layer 10-nm memristive crossbar circuits, which is only hundred times less compared to the total number of synapses in a human brain. (Reaching such extremely high integration density of synapses would also require increasing crossbar dimensions—see discussion of this point in Supplementary Note 1.)

Storing all network weights locally would eliminate overhead of the off-chip communication and lead to unprecedented system-level energy efficiency and speed for large-scale networks. For example, the crude estimates showed that energy-delay product for the inference operation of a large-scale deep learning neural networks implemented with mixed-signal circuits based on the 200-nm memristor technology similar to the one discussed in this paper could be six orders of magnitude smaller as compared to that of the advanced digital circuits, while more than eight orders of magnitude smaller when utilizing three-dimensional 10-nm memristor circuits^51^.

## Methods

### Automated forming procedure

To speed up the memristor forming, an algorithm for its automation was developed (Supplementary Fig. 1a). In general, the algorithm follows a typical manual process of applying an increasing amplitude current sweep to form a memristor. To avoid overheating during voltage controlled forming, the maximum current was limited by the current compliance implemented with external transistor connected in series with biased electrode.

In the first step of the algorithm, the user specifies a list of crossbar devices to be formed, the number of attempts, and the algorithm parameters specific to the device technology, including the initial (*I*_start_) and the final minimum (*I*_min_) and maximum (*I*_max_) values, and step size (*I*_step_) for the current sweep, the minimum current ratio (*A*_min_), measured at 0.1 V, which user requires to register successful forming, reset voltage *V*_reset_, and the threshold resistance of pristine devices (*R*_TH_), measured at 0.1 V. The specified devices are then formed, one at a time, by first checking the pristine state of the device.

In particular, if the measured resistance of as-fabricated memristor is lower than the defined threshold value, then the device is already effectively pre-formed by annealing. In this case, the forming procedure is not required, and the device is switched into the low conducting state to reduce leakage currents in the crossbar during the forming of the subsequent devices from the list.

Alternatively, a current sweep (or voltage) is applied to the device to form the device. If forming is failed, the amplitude of the maximum current in a sweep is increased and the process is repeated. (The adjustment of the maximum sweep current is performed manually in this work but could be easily automated as well.) If the device could not be formed within allowed number of attempts, the same forming procedure is performed again after resetting all devices in the crossbar to the low conductive states. The second try could still result in successful forming, if the failure to form in the first try was because of large leakages via on-state memristors that were already formed. Even though all formed devices are reset immediately after forming, some of them may be accidentally turned on during forming of other devices. Finally, if a device could not be formed within allowed number of attempts for the second time, it is recorded as defective.

### Experimental setup

Supplementary Fig. 2 shows additional details of the MLP implementation and the measurement setup. We have used AD8034 discrete operational amplifiers for the CMOS-based neurons and ADG1438 discrete analog multiplexers to implement on-board switch matrix.

### Data availability

The data that support the plots within this paper and other findings of this study are available from the corresponding author upon reasonable request.

## Electronic supplementary material

Supplementary Information
