# Supplementary material for: Implementation of multilayer perceptron network with highly uniform passive memristive crossbar circuits
Source: Nat Commun. 2018 Jun 13;9:2331. doi: 10.1038/s41467-018-04482-4 (PMC5998062; doi:10.1038/s41467-018-04482-4)
Supplement: Supplementary file 1 — Supplementary Information [file 41467_2018_4482_MOESM1_ESM.pdf]

Supplementary Information

Supplementary Figures

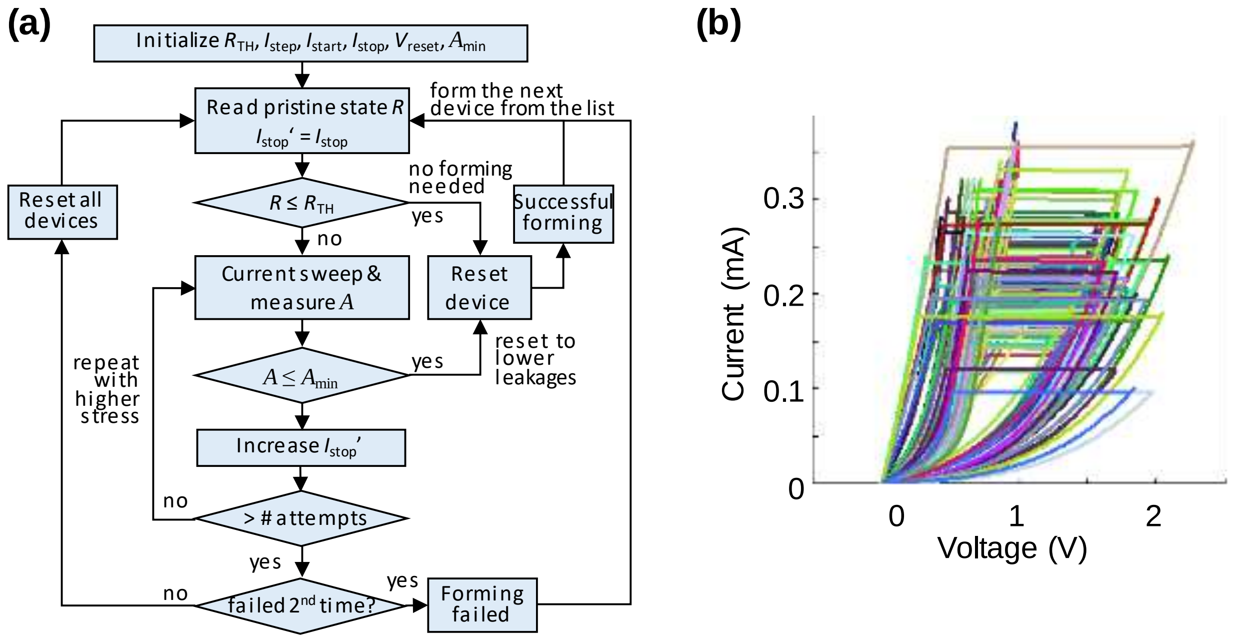

Supplementary Figure 1. **Forming procedure.** (a) Flow diagram of the automatic current-controlled memristor forming procedure. (In the voltage-controlled forming algorithm, currents should be replaced with corresponding voltages.) The adjustment of I_{stop}' value was so far performed manually after the failure to form a device automatically (in ~10% of all cases). (b) All forming *I*-*V* curves for one of the crossbars used in the experimental demonstration (with I_{start} = 180 µA, I_{stop} = 540 µA, I_{step} = 20 µA, V_{reset} = -1.3 V, A_{min} = 5).

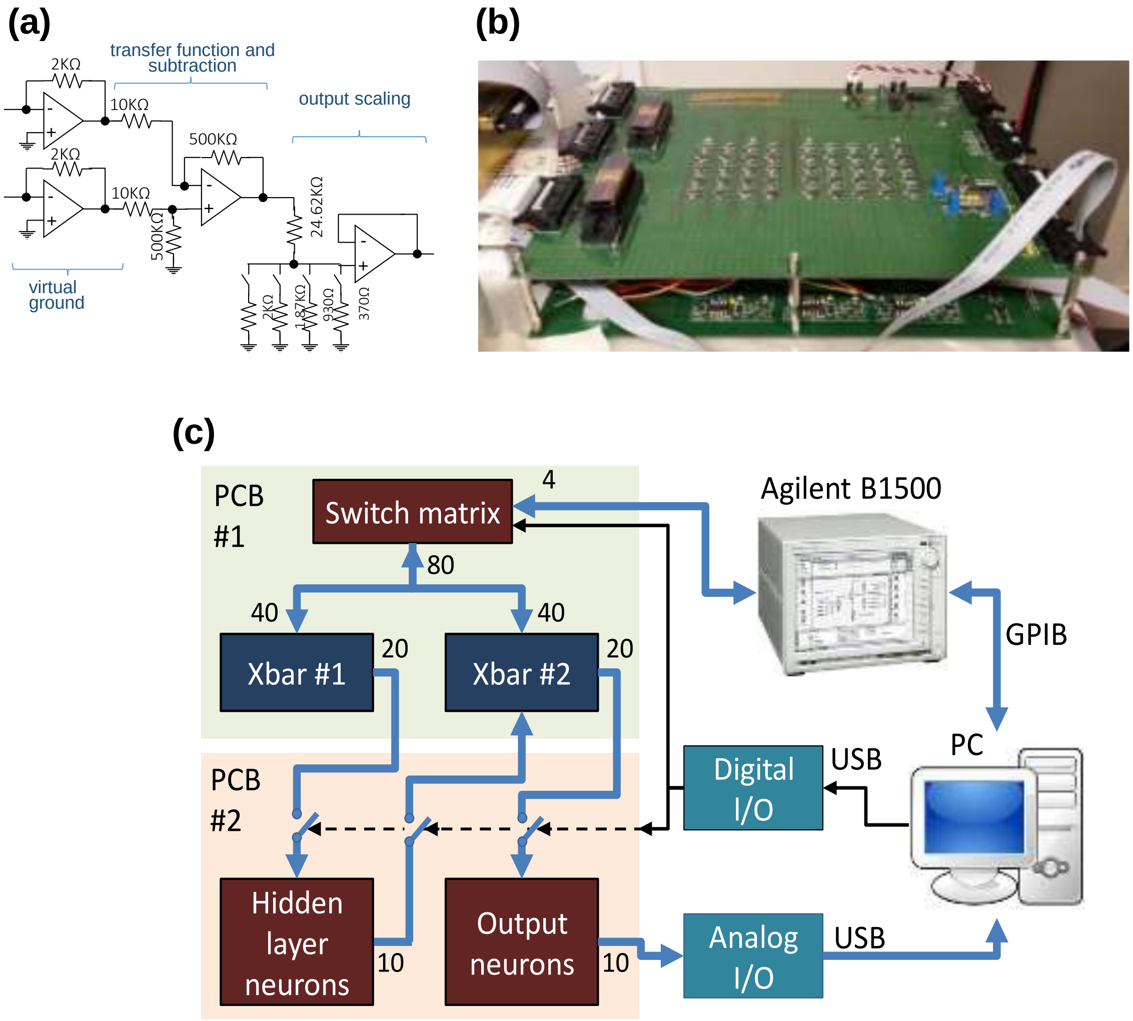

Supplementary Figure 2. Experimental setup and board details. (a) Circuit diagram of the implemented neurons. Note that the output scaling stage is not implemented in the output neurons; (b) Photos of the two printed circuit boards with one hosting wire-bonded memristive crossbar chips and the switching matrix and the other one implementing discrete CMOS neurons; (c) Block diagram of the experimental setup controlled by a personal computer.

(a)

5.25E+04	8.57E+04	8.64E+04	6.94E+04	5.98E+04	6.67E+04	7.69E+04	6.27E+04	6.41E+04	5.38E+04	6.24E+04	5.09E+04	6.16E+04	6.65E+04	8.61E+04	7.38E+04	1.17E+05	9.41E+04	8.83E+04	8.63E+04
8.11E+04	8.82E+04	6.74E+04	5.61E+04	6.98E+04	7.46E+04	7.93E+04	7.72E+04	7.73E+04	8.86E+04	6.80E+04	8.00E+04	7.43E+04	5.64E+04	6.98E+04	8.82E+04	8.45E+04	8.23E+04	9.70E+04	9.80E+04
8.49E+04	6.98E+04	5.67E+04	7.81E+04	8.27E+04	5.99E+04	8.53E+04	8.80E+04	9.02E+04	9.18E+04	9.08E+04	4.01E+04	1.02E+05	6.96E+04	5.93E+04	7.15E+04	1.04E+05	8.01E+04	1.00E+05	9.85E+04
6.64E+04	5.91E+04	4.94E+04	8.78E+04	7.73E+04	5.11E+04	6.15E+04	7.21E+04	8.69E+04	8.51E+04	5.88E+04	5.06E+04	8.01E+04	8.15E+04	6.56E+04	5.91E+04	6.37E+04	9.41E+04	9.23E+04	8.36E+04
6.12E+04	6.36E+04	7.92E+04	7.54E+04	6.19E+04	1.51E+04	2.37E+04	5.29E+04	8.68E+04	5.93E+04	2.41E+04	1.72E+04	5.11E+04	8.79E+04	7.48E+04	5.67E+04	5.74E+04	9.19E+04	9.18E+04	9.22E+04
6.11E+04	7.11E+04	7.72E+04	7.59E+04	6.45E+04	1.49E+04	1.66E+05	5.74E+04	7.23E+04	5.78E+04	1.85E+04	8.50E+04	6.47E+04	7.15E+04	7.46E+04	6.65E+04	7.36E+04	9.08E+04	1.20E+05	9.02E+04
5.20E+04	6.65E+04	6.48E+04	7.73E+04	4.93E+04	1.49E+04	1.68E+04	5.16E+04	8.26E+04	5.25E+04	1.68E+04	1.65E+04	5.23E+04	7.45E+04	7.16E+04	6.69E+04	7.15E+04	6.22E+04	8.80E+04	1.03E+05
6.23E+04	6.46E+04	6.52E+04	1.01E+05	5.82E+04	2.46E+04	3.27E+04	5.65E+04	9.71E+04	6.47E+04	3.75E+04	2.39E+04	5.46E+04	5.46E+04	6.46E+04	6.94E+04	1.04E+04	6.52E+04	6.78E+04	9.76E+04
6.12E+04	6.74E+04	6.96E+04	7.05E+04	1.15E+05	5.51E+04	6.09E+04	7.04E+04	7.18E+04	7.06E+04	5.81E+04	6.48E+04	6.91E+04	6.80E+04	7.12E+04	8.19E+04	6.11E+04	6.37E+04	8.58E+04	9.45E+04
5.88E+04	5.73E+04	4.21E+04	3.67E+04	4.23E+04	5.20E+04	6.27E+04	5.93E+04	4.35E+04	6.03E+04	6.66E+04	5.07E+04	4.29E+04	3.39E+04	4.98E+04	6.12E+04	7.29E+04	5.13E+04	9.06E+04	9.24E+04
5.50E+04	7.23E+04	2.58E+04	6.81E+03	2.68E+04	3.20E+04	4.69E+04	4.23E+04	5.41E+04	4.40E+04	4.10E+04	4.22E+04	2.39E+04	7.26E+03	2.52E+04	6.02E+04	5.80E+04	6.77E+04	7.91E+04	1.00E+05
6.23E+04	6.57E+04	4.41E+04	1.55E+04	4.75E+04	5.52E+04	5.16E+04	5.76E+04	5.27E+04	5.10E+04	5.17E+04	5.41E+04	4.23E+04	1.64E+04	4.28E+04	7.65E+04	6.11E+04	6.40E+04	8.15E+04	1.01E+05
5.68E+04	6.07E+04	5.14E+04	1.67E+04	7.33E+03	1.47E+04	3.18E+04	4.36E+04	4.41E+04	3.29E+04	3.42E+04	1.66E+04	6.73E+03	2.49E+04	4.39E+04	6.06E+04	6.06E+04	5.41E+04	8.07E+04	9.32E+04
6.13E+04	4.91E+04	6.84E+04	4.31E+04	2.41E+04	1.59E+04	2.57E+04	2.53E+04	2.57E+04	2.40E+04	1.58E+04	1.52E+04	2.31E+04	4.99E+04	7.17E+04	5.31E+04	5.52E+04	8.37E+04	9.48E+04	1.04E+05
8.02E+04	5.25E+04	6.43E+04	6.55E+04	6.20E+04	3.63E+04	2.65E+04	2.74E+04	2.02E+04	2.62E+04	2.46E+04	4.34E+04	6.50E+04	6.27E+04	6.06E+04	5.18E+04	7.26E+04	1.01E+05	9.68E+04	9.53E+04
8.91E+04	9.28E+04	5.30E+04	6.31E+04	7.11E+04	6.17E+04	5.86E+04	4.75E+04	4.86E+04	5.12E+04	5.68E+04	6.37E+04	7.19E+04	5.06E+04	4.26E+04	7.45E+04	1.03E+05	1.07E+05	9.84E+04	8.63E+04
9.10E+04	7.95E+04	7.98E+04	5.75E+04	5.40E+04	5.81E+04	6.39E+04	6.86E+04	7.50E+04	7.59E+04	6.31E+04	6.09E+04	5.35E+04	8.79E+04	7.82E+04	9.04E+04	1.04E+05	9.93E+04	9.81E+04	1.02E+05
1.16E+05	9.55E+04	7.54E+04	8.55E+04	6.97E+04	6.62E+04	5.13E+04	4.93E+04	5.09E+04	4.98E+04	4.91E+04	6.05E+04	7.06E+04	8.32E+04	9.28E+04	9.09E+04	9.24E+04	9.68E+04	9.82E+04	9.29E+04
9.12E+04	9.31E+04	9.29E+04	9.51E+04	1.13E+05	4.21E+04	6.91E+04	5.33E+04	6.53E+04	8.10E+04	7.55E+04	8.94E+04	1.11E+05	9.26E+04	1.33E+05	8.92E+04	9.39E+04	9.37E+04	9.10E+04	9.40E+04
8.44E+04	9.53E+04	9.60E+04	8.73E+04	8.32E+04	8.22E+04	8.23E+04	1.71E+07	9.20E+04	5.01E+06	7.97E+04	6.19E+04	9.14E+04	9.66E+04	9.17E+04	9.28E+04	1.00E+05	9.72E+04	8.26E+04	7.31E+04

(b)

4.41E+04	1.09E+04	1.02E+04	6.25E+02	1.14E+03	3.27E+03	-6.92E+03	1.57E+04	1.43E+04	2.46E+04	7.63E+03	1.91E+04	-7.33E+02	3.48E+03	1.05E+04	2.28E+04	-2.00E+04	2.46E+03	8.34E+03	1.03E+04
1.55E+04	-6.81E+02	2.62E+03	4.82E+03	1.86E+02	3.77E+03	8.22E+03	1.03E+04	1.02E+04	-1.07E+03	1.95E+04	-1.64E+03	-4.32E+03	4.53E+03	1.81E+02	-7.16E+02	1.21E+04	1.43E+04	-4.09E+02	-1.41E+03
2.61E+03	1.72E+02	4.24E+03	-8.08E+03	4.78E+03	2.76E+04	2.18E+03	-4.87E+02	-2.70E+03	-4.30E+03	-3.27E+03	4.74E+04	-1.47E+04	3.62E+02	1.58E+03	-1.52E+03	-7.37E+03	1.65E+04	-3.45E+03	-1.93E+03
3.57E+03	1.78E+03	2.06E+04	-2.55E+02	1.08E+03	7.31E+02	-5.66E+02	6.29E+03	6.18E+02	-6.72E+03	2.12E+03	1.16E+03	-1.66E+03	5.98E+03	4.42E+03	1.82E+03	6.26E+03	2.50E+03	4.26E+03	1.30E+04
-3.25E+02	-2.71E+03	-7.98E+02	2.97E+03	-9.54E+02	1.01E+03	1.47E+03	7.98E+03	6.97E+02	1.55E+03	1.15E+03	-1.09E+03	9.75E+03	-9.55E+03	3.59E+03	4.19E+03	3.46E+03	-4.45E+03	4.80E+03	4.39E+03
-2.45E+02	-1.14E+03	1.18E+03	2.49E+03	-3.56E+03	1.15E+03	-6.96E+04	3.49E+03	6.10E+03	3.10E+03	-2.45E+03	1.16E+04	-3.85E+03	6.92E+03	3.80E+03	3.52E+03	-1.27E+04	-2.08E+04	-3.25E+04	6.43E+03
8.92E+03	3.48E+03	5.15E+03	1.12E+03	2.53E+03	1.18E+03	-6.60E+02	1.68E+02	-4.17E+03	-6.57E+02	-6.96E+02	-3.91E+02	-4.80E+02	3.88E+03	-1.57E+03	3.10E+03	-1.06E+04	-1.31E+03	-4.55E+02	-6.04E+03
-1.36E+03	5.40E+03	4.76E+03	-3.11E+04	2.68E+03	5.50E+02	8.74E+02	4.44E+03	-2.71E+04	-3.81E+03	-3.90E+03	1.28E+03	6.25E+03	1.54E+04	5.39E+03	5.63E+02	5.05E+04	-4.30E+03	1.97E+04	-9.76E+02
-2.55E+02	2.58E+03	4.13E+02	-5.33E+02	-4.53E+04	-3.34E+03	4.60E+00	-3.94E+02	-1.79E+03	-6.47E+02	2.80E+03	-3.90E+03	9.27E+02	2.05E+03	-1.20E+03	-1.19E+04	-1.58E+02	-2.84E+03	1.67E+03	2.07E+03
2.06E+03	3.57E+03	5.67E+02	-3.06E+03	3.66E+02	-1.65E+02	-1.79E+03	1.58E+03	1.74E+04	5.80E+02	-5.70E+03	1.11E+03	-2.36E+02	-2.56E+02	2.05E+03	-2.75E+02	-1.20E+04	5.41E+02	-3.13E+03	4.23E+03
5.86E+03	-1.14E+04	-6.28E+02	1.87E+02	-1.64E+03	1.63E+03	-4.23E+03	4.18E+02	-2.32E+03	-1.34E+03	1.67E+03	-8.59E+03	1.29E+03	-2.57E+02	1.26E+01	7.47E+02	2.85E+03	-6.81E+03	8.42E+03	-3.68E+03
-1.43E+03	-4.76E+03	-1.40E+03	6.16E+02	-4.84E+03	-3.43E+03	1.98E+02	-5.79E+03	-9.21E+02	7.97E+02	1.43E+02	-2.29E+03	4.05E+02	-3.09E+02	-6.93E+01	-6.50E+03	-1.93E+02	-3.14E+03	6.05E+03	-4.14E+03
4.07E+03	1.75E+02	4.24E+02	-5.82E+02	-3.31E+02	1.37E+03	1.77E+03	-9.17E+02	-1.39E+03	7.29E+02	-6.46E+02	-4.84E+02	2.66E+02	3.10E+02	7.89E+03	2.98E+02	2.95E+02	2.43E+04	6.81E+03	3.44E+03
-3.71E+02	2.75E+03	1.56E+03	8.70E+03	1.12E+03	2.08E+02	-4.85E+02	-1.40E+02	-5.06E+02	1.21E+03	2.89E+02	8.73E+02	2.10E+03	1.95E+03	-1.69E+03	-1.33E+03	5.67E+03	3.82E+03	1.80E+03	-7.07E+03
-1.77E+03	-6.81E+02	-3.44E+03	4.48E+03	-1.06E+03	6.40E+03	-1.33E+03	-2.17E+03	4.95E+03	-1.03E+03	5.86E+02	-7.48E+02	-4.12E+03	7.34E+03	3.16E+02	-2.26E+01	5.84E+03	-4.04E+03	-1.92E+02	1.34E+03
7.54E+03	-1.44E+04	-1.17E+03	-2.24E+03	-1.11E+03	-8.14E+02	2.29E+03	4.35E+03	3.20E+03	5.80E+02	4.12E+03	-2.84E+03	-1.91E+03	1.19E+03	9.16E+03	3.92E+03	-5.97E+03	-1.02E+04	-1.79E+03	1.03E+04
5.56E+03	1.71E+04	-1.39E+03	3.41E+03	-2.18E+03	2.79E+03	-3.04E+03	1.39E+03	-4.96E+03	-5.95E+03	-2.23E+03	-1.60E+01	-1.69E+03	-2.70E+04	1.56E+02	6.19E+03	-7.13E+03	-2.68E+03	-1.49E+03	-5.12E+03
-1.98E+04	1.10E+03	2.12E+04	2.03E+03	3.21E+02	-5.27E+03	5.36E+02	2.46E+03	8.85E+02	1.96E+03	2.66E+03	4.14E+02	-5.79E+02	4.25E+03	3.79E+03	5.69E+03	4.21E+03	-1.84E+02	-1.60E+03	3.72E+03
5.41E+03	3.49E+03	3.72E+03	1.54E+03	-1.60E+04	4.54E+04	9.34E+03	1.67E+04	4.72E+03	-1.10E+04	2.92E+03	-1.86E+03	-1.42E+04	4.02E+03	-3.65E+04	7.37E+03	2.74E+03	2.94E+03	5.63E+03	2.60E+03
1.22E+04	1.34E+03	6.22E+02	9.32E+03	1.34E+04	5.29E+03	5.21E+03	-1.70E+07	-4.49E+03	-4.92E+06	7.80E+03	2.56E+04	5.24E+03	-5.00E-01	4.94E+03	3.84E+03	-3.79E+03	-5.92E+02	1.40E+04	2.35E+04

Supplementary Figure 3. **Results for smiley face tuning experiment.** (a) Absolute device resistances and (b) absolute tuning error.

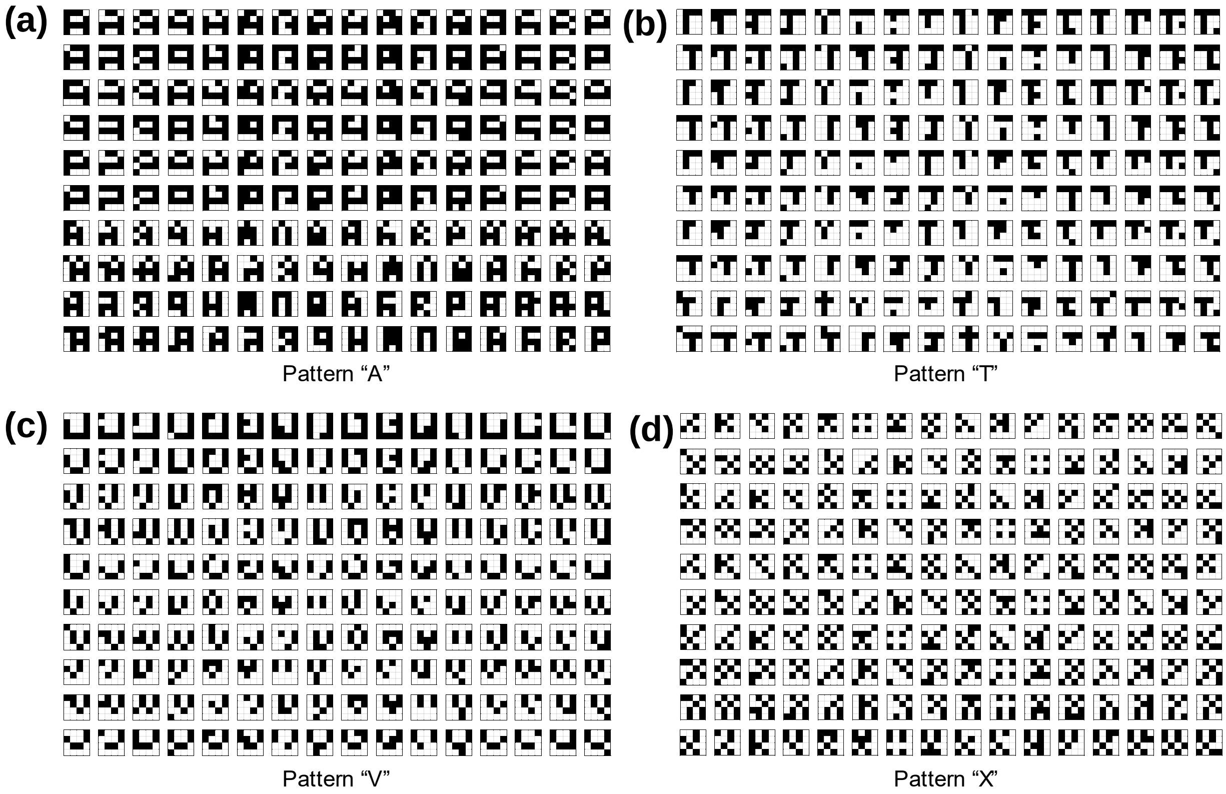

Supplementary Figure 4. Pattern classification test set. (a-d) A complete set of 640 test patterns for four letters used in the pattern classification experiment.

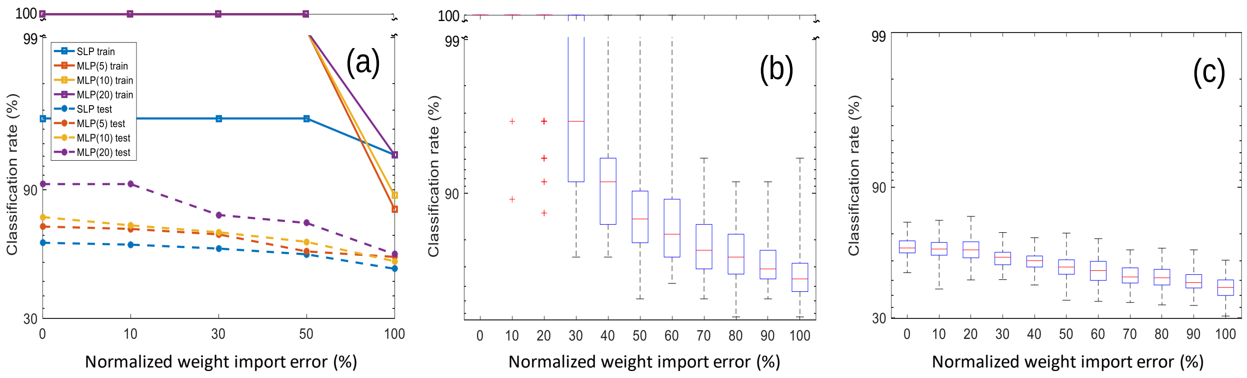

Supplementary Figure 5. Perceptron software simulation results. (a) Comparison of the best fidelity obtained for single layer perceptron and MLPs with different number of hidden layer neurons (shown in parenthesis in the legend). (b, c) The results for 10-hidden layer perceptron, similar to the one used in the experiment for classification of (b) training and (c) test patterns. The normalized weight import error (Error) was modeled by using a random variate generated from uniform distribution [W_{ideal} - W_{ideal}*Error/100, W_{ideal} + W_{ideal}* Error/100], where W_{ideal} is the desired weight value. Such import error approach approximates well the resulting conductance distribution for relatively crude tuning accuracy, e.g. 30% that was used in our experiment. The red, blue (rectangles), and black (segment) markers denote, respectively, the median, the 25%-75% percentile, and the minimum and maximum values for 100 simulation runs.

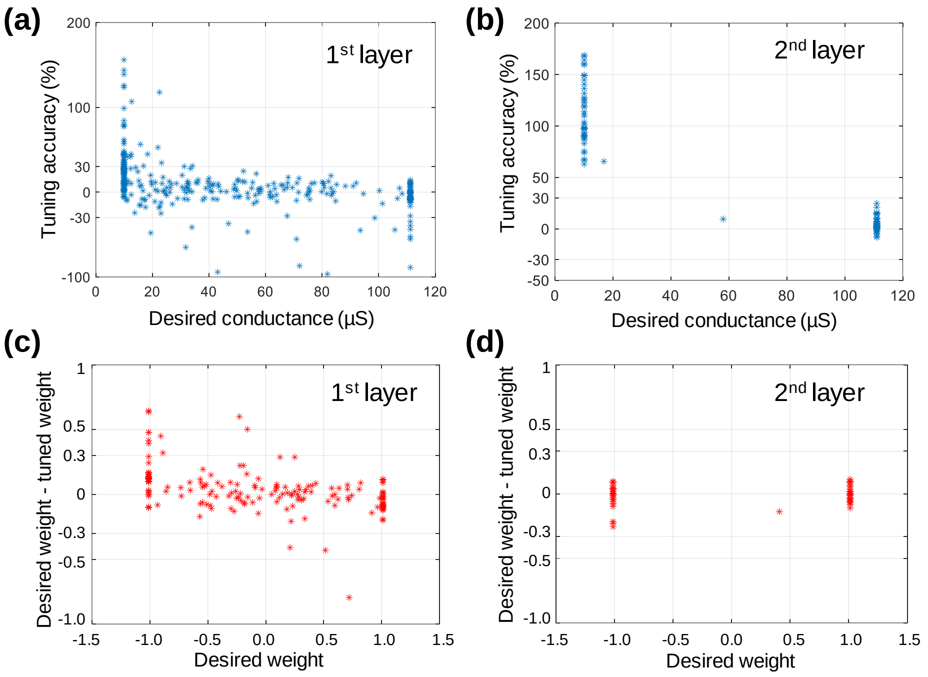

Supplementary Figure 6. Tuning results for classifier experiment. (a, b) Tuning accuracy and (c, d) weight errors for each of the two layers of the implemented MLP network. The data for tuning accuracy are replotted from Fig. 5a,b of the main text. The tuning accuracy is defined here again as the normalized difference between the desired and actual conductances. The shown weight error is calculated as a (not normalized) difference between the desired value and the actual one implemented with the pair of memristors. Note that the weights and conductances in the second layer are always close to their maximum or minimum values, because of the clipping enforced during software ex-situ training.

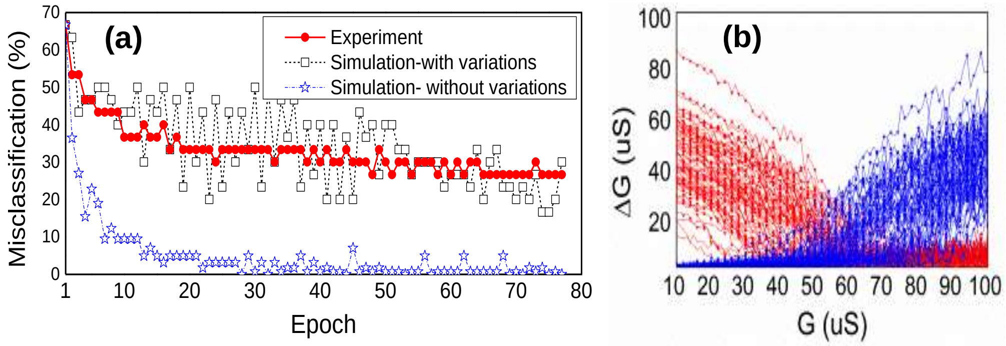

Supplementary Figure 7. In-situ training for 3-pattern classification ('A', 'V', and 'T'). (a) Experimentally measured and simulated error decay dynamics for the training set patterns. In experiment, conductances of all memristors were updated, one row of the crossbar at a time, at the end of each epoch. The weight update in each row was done in parallel in two steps by applying 500-µs fixed amplitude (± 1.3 V) voltage pulses using *V*/2 biasing technique. (b) Example of devices' switching kinetics and it's variations obtained using simple device model from Ref. [1]. Such model was used for the in-situ training simulations shown in panel a – see supplementary matlab code for more details.

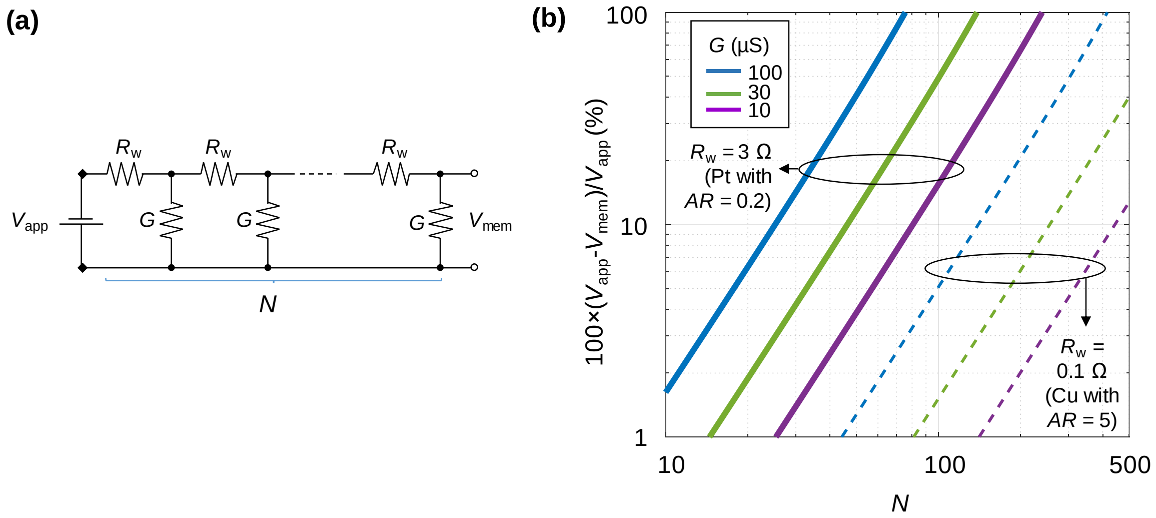

Supplementary Figure 8. Voltage drop in resistor ladder. (a) The considered circuit and (b) the relative worst-case voltage drop for several representative parameters specific to the implemented crossbar circuits. AR stands for the electrode height-to-width aspect ratio.

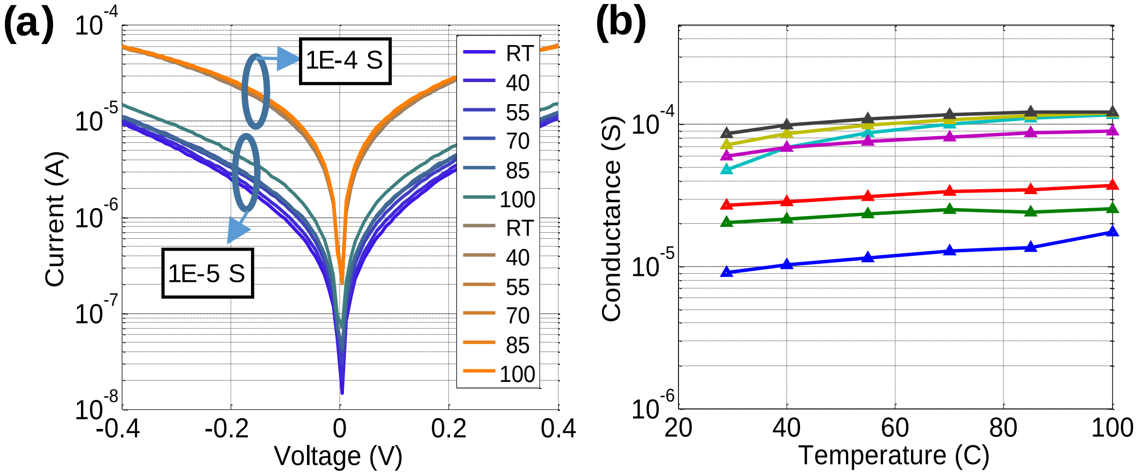

Supplementary Figure 9. Temperature sensitivity. (a) The *I-V* curves of a single memristor for several temperatures and (b) the extracted temperature dependence of its conductance.

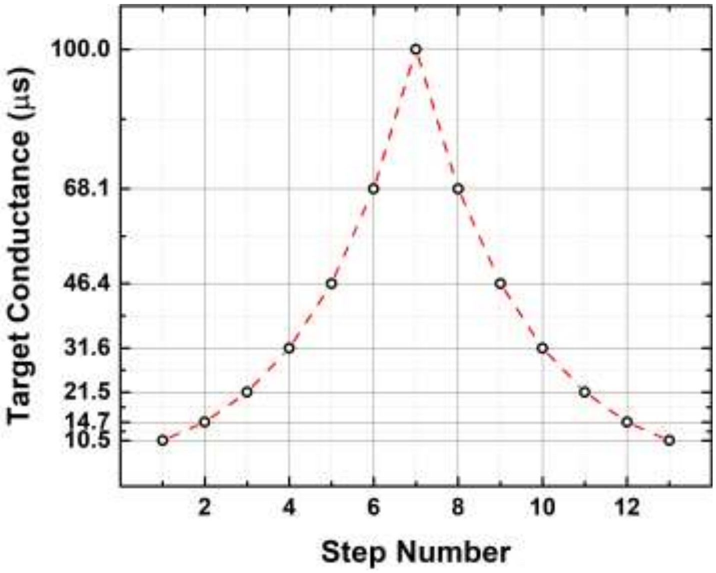

Supplementary Figure 10. Target conductances for additional tuning experiment. The sequence of target conductance values, exponentially spaced between 10.5 μS and 100 μS, that were used in the additional tuning experiment.

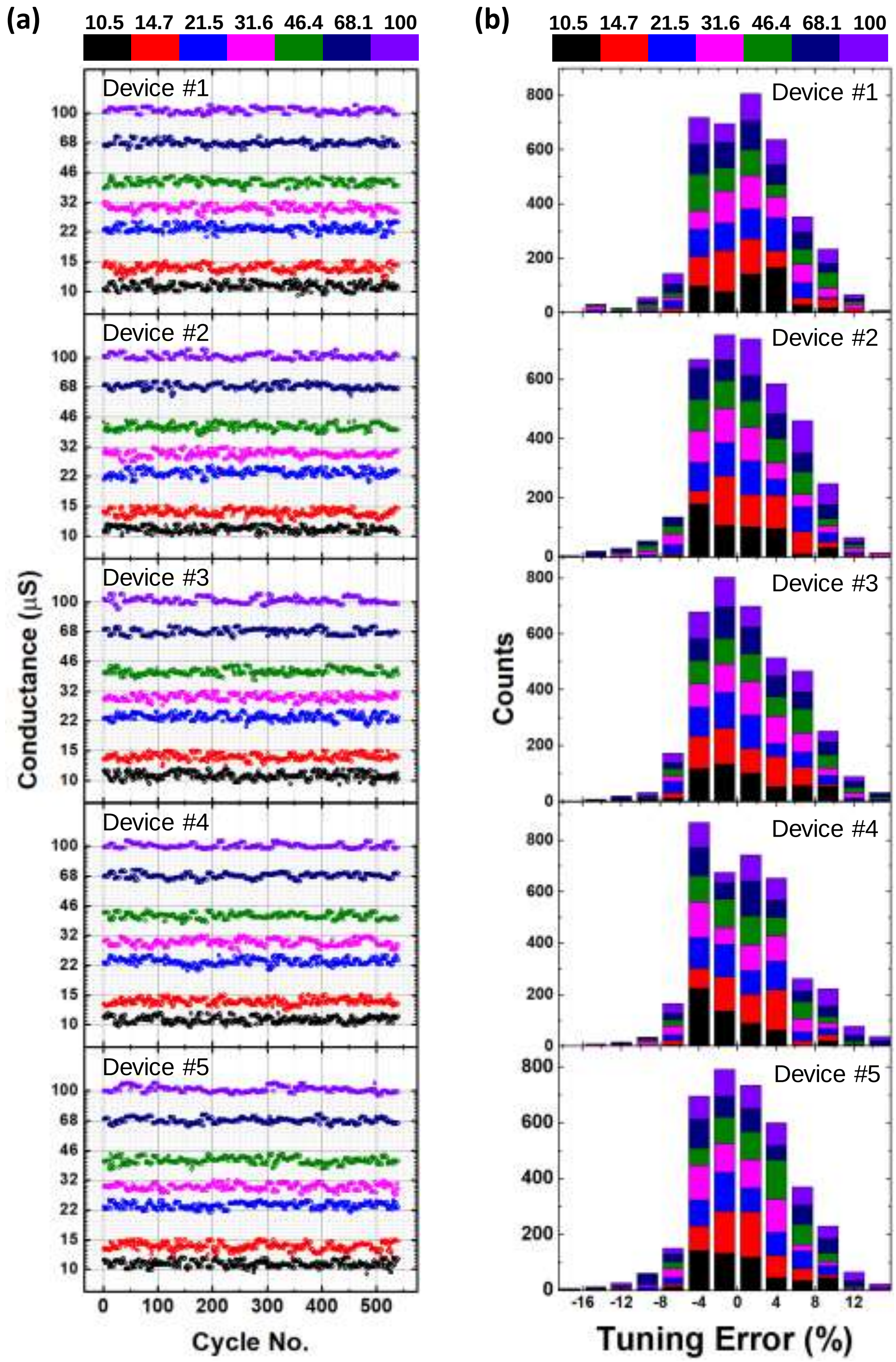

Supplementary Figure 11. Experimental results for repeated tuning. The data are shown for 5 crossbar integrated memristors. (a) Each dot shows final measured value for the tuned conductance. One cycle corresponds to tuning of all 5 memristors to 13 specific conductance values, as shown in Supplementary Figure 10. (b) Corresponding tuning error histogram, shown separately for each device. The tuning error is defined as a normalized difference between the desired and actual conductance. Bins are 2.67 % wide.

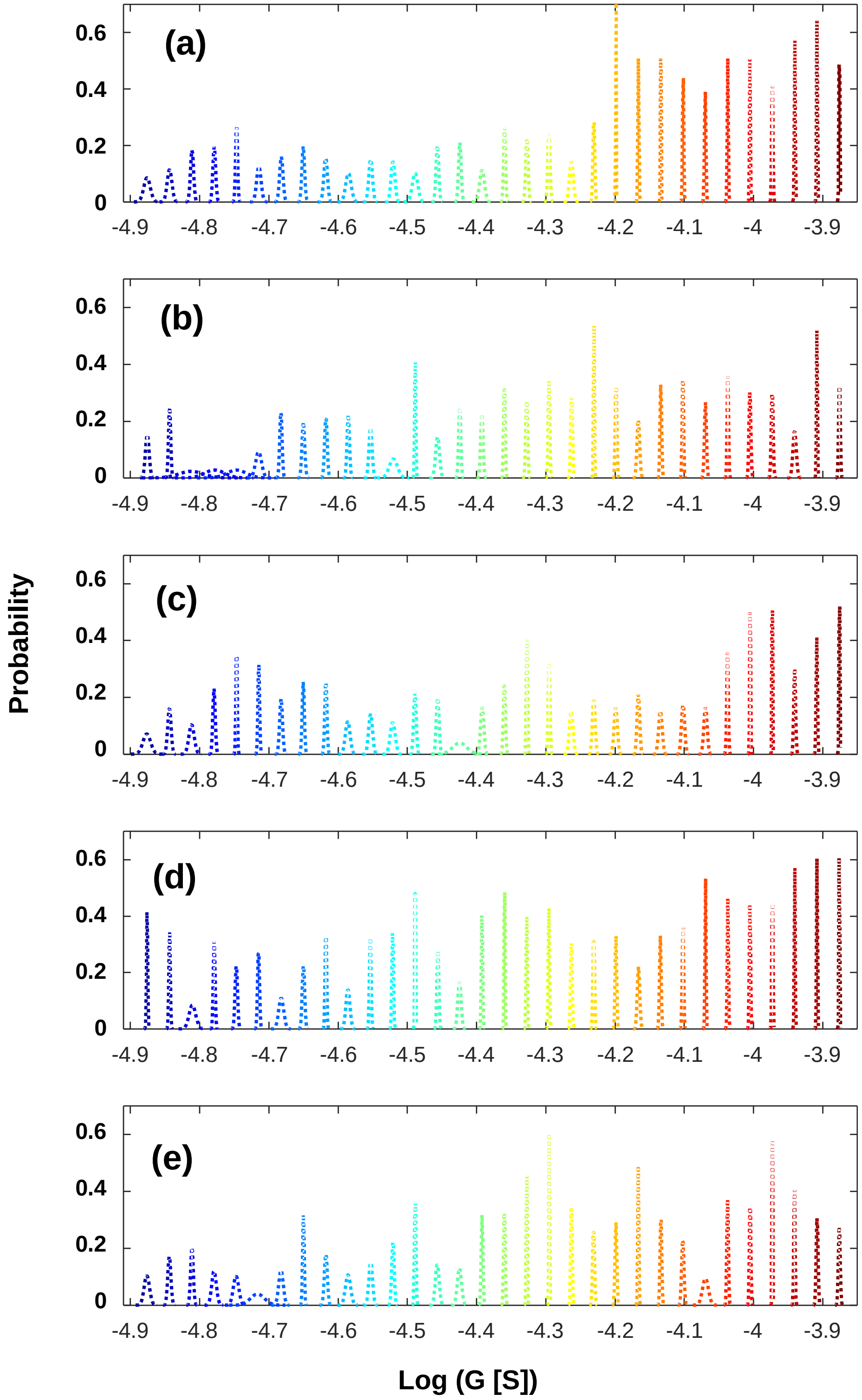

Supplementary Figure 12. Experimental results for high-precision tuning. (a-e) The data shows the results of tuning conductances of 5 crossbar integrated memristors to 32 exponentially spaced levels within 7.5-75 kΩ range (at 0.2 V) with 2.5% tuning accuracy. Each panel shows histograms of tuning the same memristors 20 times to each level. The dashed lines are normal fits for the experimental data.

Supplementary Note 1: Crossbar Circuit Scaling

An important future work, in addition to the monolithic integration with CMOS subsystem discussed in the main text, is increasing the dimensions of the crossbar circuits which would allow higher connectivity among neurons and improve integration density (i.e. by lowering relative peripheral overhead). Here let us first stress again that in our implementation, crossbar lines are never floated so that sneak path currents do not affect directly the measured currents at the outputs. Scaling up crossbar dimensions, however, increases currents flowing in the crossbar lines. Because of the potential voltage drops across the crossbar lines the voltages applied to the crosspoint memristors could be different from the ones applied at the periphery.

For example, Supplementary Figure 8 shows the dependence of the worse-case voltage drop as a function of the length of the finite resistor ladder, which is useful for analyzing crossbar circuit operation. In this figure, one set of lines shows the voltage drop assuming electrode resistance per wire segment (R_w) comparable to the one in our experiment, while the other one is for more aggressive (though quite realistic) parameters which are representative of high-aspect ratio copper wires. For simplicity, the memristor conductances $G(V)$ can be estimated using the corresponding average value measured at bias V, specific to the type of considered operation. It should be noted that in a properly trained network, the weights are typically normally distributed so that the representative average value is rather close to the minimum of the used conductance range.

Let us now consider in detail three operations which might be impacted by voltage drop on the crossbar lines, namely classifier inference, and read and write phases of the tuning algorithm:

Write operation

Naturally, the voltage drops are the most significant for write operation because of the larger voltages applied and higher currents passed. For the conductance tuning, however, we do not rely on precise conductance update with write pulses but rather adjust applied write voltages gradually based on precise read measurements. Therefore, any potential voltage drop will be compensated dynamically during tuning by applying larger voltage pulses, with the largest applied voltage (and hence crossbar dimensions) limited by the condition of not disturbing half-selected devices.

Specifically, let us assume the $V/3$ biasing scheme, i.e. with $\pm V_W/2$ applied to the selected lines and $\pm V_W/6$ to the remaining lines. From Fig. 1c and 2, up to $(V_{TH}^{SET})_{max} \approx +1.3$ V set and

$(V_{TH}^{RESET})_{max} \approx$ -1.9 V reset voltages must be applied to switch the devices with the largest switching thresholds. (Here, we neglect the tails of the distributions on Fig. 2, which are typically contributed by the devices at the edges of the array. This is similar to the dummy line technique commonly used in conventional memories.) The corresponding average memristor conductances at one third of such biases can be roughly estimated to be $\langle G((V_{TH}^{SET})_{max}/3)\rangle \approx 30$ μS for set and $\langle G((V_{TH}^{SET})_{max}/3)\rangle \approx 50$ μS for reset transitions. On the other hand, the largest voltages, which can be safely applied to the half-selected devices without disturbing memristors with the smallest switching thresholds are $(V_{TH}^{SET})_{min} \approx +0.7$ V for set and $(V_{TH}^{RESET})_{min} \approx -1$ V for reset transitions. The maximum crossbar dimensions, specific to the wire resistance, memristor *I-V* and its variations (i.e. parameters R_w, $G((V_{TH})_{max}/3)$, $(V_{TH})_{max/min}$) can be crudely estimated assuming $100\times(3(V_{TH})_{min} - (V_{TH})_{max})/(V_{TH})_{max} / 2$ as the largest allowable relative voltage drop in Supplementary Figure 8b. (Additional factor of 2 in the denominator accounts for the drop on both selected lines.) For the considered parameters, this drop is equal to 30% and 25% for set and reset switching, respectively, indicating to the possibility of implementing 70×70 crossbar arrays with demonstrated device technology and up to 400×400 crossbar array for the crossbar arrays with improved electrode resistance. (Note that in our work, we have used somewhat simpler, the *V*/2 biasing scheme, for which the largest allowable voltage drop is ~ 7% and the corresponding maximum crossbar dimensions are around 40×40 and 200×200 for two considered electrode resistances.)

Read operation

Let us assume that during read operation, one of the selected lines is biased at $+V_R$, while the other selected line and all of the remaining ones are grounded. (This is exactly the scheme that we used for conductance tuning in this work.) In this case, the current running via grounded selected crossbar line is small (only contributed by one selected memristor) and does not dependent on the crossbar dimensions. Therefore, the substantial voltage drops may occur only on the biased selected crossbar line. Such voltage drop would be naturally much less than that of the write operation and, moreover, it can be easily taken into account when reading the state of the devices. For example, it is straightforward to compute the actual applied voltage across the specific memristor knowing the conductive states of all other half-selected devices of the biased selected crossbar electrode.

Inference operation

As discussed in main text, during inference, one set of lines (vertical in Figure 3a) receive voltages $V \leq V_R$, while all orthogonal lines are virtually grounded. Because of the smaller applied voltages, the crossbar line currents, and hence the corresponding voltage drops, are the smallest for inference operations. However, the inference operation (just like read) is more sensitive as compared to write operation to the voltage variations and even small voltage drops may lead to the lower effective precision of the vector-by-matrix computation. For example, assuming representative 10 μS average device conductance, and 70×70 and 400×400 crossbar arrays discussed in write operation above, the worst-case voltage drop on one line is around 7% (Supplementary Figure 8b).

Using our examples, inference operations would likely be a limiting factor for scaling though are several reserves for improvements. For example, the conductances of each memristor can be uniquely increased to compensate for the potential voltage drops during inference. (Unlike read operation, such adjustment cannot be exact because of the input-dependent voltage drop on the virtually-grounded lines.) The loss of precision for the worst case largest currents might be also acceptable, e.g. if it leads to the saturation of the neuron. It is also important to note that precision loss at inference due to voltage drops is common problem for the devices with or without selectors. If fact, the problem is likely more severe for 1T1R structures, because of their larger device area and potentially larger R_w.

The crude estimate above show that the developed device technology, with some further optimization of the electrodes, should be suitable for implementing much larger, up to 400×400 crossbar circuit. The discussed analysis is also applicable to 10 nm memristors, if we assume that both the resistance of the crossbar line segment and memristor operating (average) currents would scale down at the same rate. (For that memristor currents should decrease at slightly faster rate than its linear device dimensions to compensate for the additional increase in metal resistivity due to scattering effects.) That is certainly plausible scenario for smaller currents at voltages below V_R (e.g., relevant to the inference operation and read phase of the tuning algorithm) considering that the off-state conductance is typically limited by the device leakages which are proportional to the device electrode area. Ensuring the same scaling in the context of the write phase of the tuning algorithm would require enhancing *I*-*V* nonlinearity and/or decreasing write currents, which we believe is also plausible given the observed write current dependence on the electrode area in our devices and further optimization of the tunneling barrier layer.

Supplementary Note 2: Device Programmability and Uniformity

We have performed a number of additional experiments to characterize device to device variations in tunability. In the first experiment, we have repeatedly tuned 5 crossbar integrated devices to the same set of conductance levels. Specifically, in one cycle each device was sequentially tuned to 13 exponentially-spaced values within 10.5-100 μS range of conductances (measured at 0.2 V), which is a typical operating range utilized during inference computation. The first target conductance value was 10.5 μS. It was then increased to 100 μS in 6 steps before decreasing it back to 10.5 μS, also in 6 steps (Supplementary Figure 10). Such tuning cycle was repeated about 550 times in the same order for every device. For the tuning algorithm, the write pulse polarity and magnitudes were selected according to the tuning algorithm described in Ref. 2. We used 0.2 V 100 μs read pulses with 25 μs rise and fall times. Each measured current value during read operation was an average of 10,000 samples (taken every 5 ns) within 50 μs read pulse.

The sequences of tuned conductances are presented in Supplementary Figure 11a, while the corresponding histograms for the aggregate tuning error for all devices are shown on Supplementary Figure 11b. To speed up measurements, the tuning precision was always set to 7.5%, while the maximum number of write/read pulses was set to 300. As Supplementary Figure 11 shows, in some cases the tuning accuracy was worse than the desired one due to reaching maximum number of tuning iterations. Tuning accuracy was also somewhat worse for lower values of the desired conductances, likely due to larger temporal fluctuations of read currents. The data do not show noticeable degradation in tuning accuracy over time. Note that Supplementary Figure 11a shows final values of the measured tuned conductances. Tuning to each state involved 45 write/read pulses on average, so, altogether, each device was stressed with write pulse almost 300,000 times in this experiment.

In the next experiment (Supplementary Figure 12), we tuned 5 devices with much higher, 2.5% tuning accuracy to 32 conductance levels, which were exponentially spaced within similar 7.5-75 kΩ range (at 0.2 V). Each device was tuning 20 times to each level. The data shows that most of the devices, most of the time, can be set closely to the desired states with significant margins between adjacent levels. Some of the devices at some states, however, cannot be tuned accurately. We expect that tuning accuracy would significantly improve with better control over

the shape and duration of the write pulses, which would be possible in tightly integrated CMOS/memristor circuits. Also, the infrequent nonideal behavior can be coped with various circuit and algorithmic techniques, e.g. by dynamically adjusting the conductances in differential pairs.

Supplementary References

1. Prezioso, M. *et al.* Modeling and implementation of firing-rate neuromorphic-network classifiers with bilayer $Pt/Al_2O_3/TiO_{2-x}/Pt$ memristors. In *Proc. IEEE International Electron Devices Meeting* 455-458 (2015).
2. Alibart, F. *et al.*, High-precision tuning of state for memristive devices by adaptable variation-tolerant algorithm. *Nanotechnology* **23**, 075201 (2012).
